# Bilateral Optic Disk Swelling and Peripheral Visual Field Defects as a Rare Initial Presentation of Primary Chiari I Malformation

**DOI:** 10.7759/cureus.40652

**Published:** 2023-06-19

**Authors:** Malika P Ganguli, Eric Robinson, Mahlon R Kile, David Kapp

**Affiliations:** 1 Internal Medicine, Ross University School of Medicine, Bridgetown, BRB; 2 Neurosurgery, Ross University School of Medicine, Bridgetown, BRB; 3 Internal Medicine, Norwalk Hospital, Nuvance Health, Norwalk, USA

**Keywords:** intracranial pressure, visual field defect, pseudopapilledema, optic disk swelling, monocular right lower quadrantanopia, papilledema, lumbar puncture, arnold chiari, chiari 1, chiari

## Abstract

Chiari malformation 1 (CM1) is defined as a herniation of encephalon matter through the base of the skull. The amount of herniation is cited as greater than 3 mm or 5 mm, depending on the source of literature. We report a rare case of a 55-year-old male initially presenting with bilateral papilledema and monocular right lower quadrantanopia, found to have CM1. An MRI confirmed 4.87 mm herniation of the cerebellar tonsils at the foramen magnum, and he was diagnosed with CM1. He was later found to have a normal opening pressure on lumbar puncture at 10 cm H_2_O. This poses an interesting clinical question as papilledema is defined by elevated intracranial pressure. The ophthalmic defects of this patient with normal intracranial pressure and CM1 are explored in this report.

## Introduction

Chiari malformation (CM) is a condition caused by the movement of encephalon matter outside the skull and into the spinal cord, as described by Hans Chiari in 1891 [1]. The severity of brain displacement, known as herniation, determines the classification of the malformation from type I to IV. Type I is most common and is defined by the displacement of cerebellar tonsils equal to or greater than 3 mm (pediatric) or 5 mm (adult) through the base of the skull known as the foramen magnum without the involvement of the brainstem or spinal cord [2]. Types II-IV are listed in increasing severity with variable involvement of the cerebellar vermis and tonsils, brainstem, and vertebral column and the resultant clinical sequelae. Additionally, type II is known to have an association with myelomeningocele, a type of spina bifida causing the spinal cord and meninges to herniate outside the protective encasement of the vertebral column [2]. In this paper, we will explore an abnormal presentation of optic nerve swelling in a CM1 patient with normal intracranial pressure.

## Case presentation

The patient is a 55-year-old man with a past medical history of vitiligo, resolved childhood heart murmur, and hyperlipidemia. He noticed diminished peripheral vision in the right eye and mild right-sided headache over the parietal and apex of his head after a hike. The headache later resolved. Both an optometrist and an ophthalmologist diagnosed him with papilledema, with the right eye showing more pronounced symptoms. On presentation, he denied signs of increased intracranial pressure, including nausea, vomiting, and dizziness. He also reported no changes in hearing or coordination and had no history of trauma. Neurological evaluation revealed right monocular right lower quadrantanopia without other focal deficits. Color vision was intact, and his mental status was normal. Cranial nerves II-XII were normal, with no pronator drift, full strength throughout, normal light touch, normal finger-to-nose testing, and alternating hand movements. The patient did not have scoliosis.

**Figure 1:**
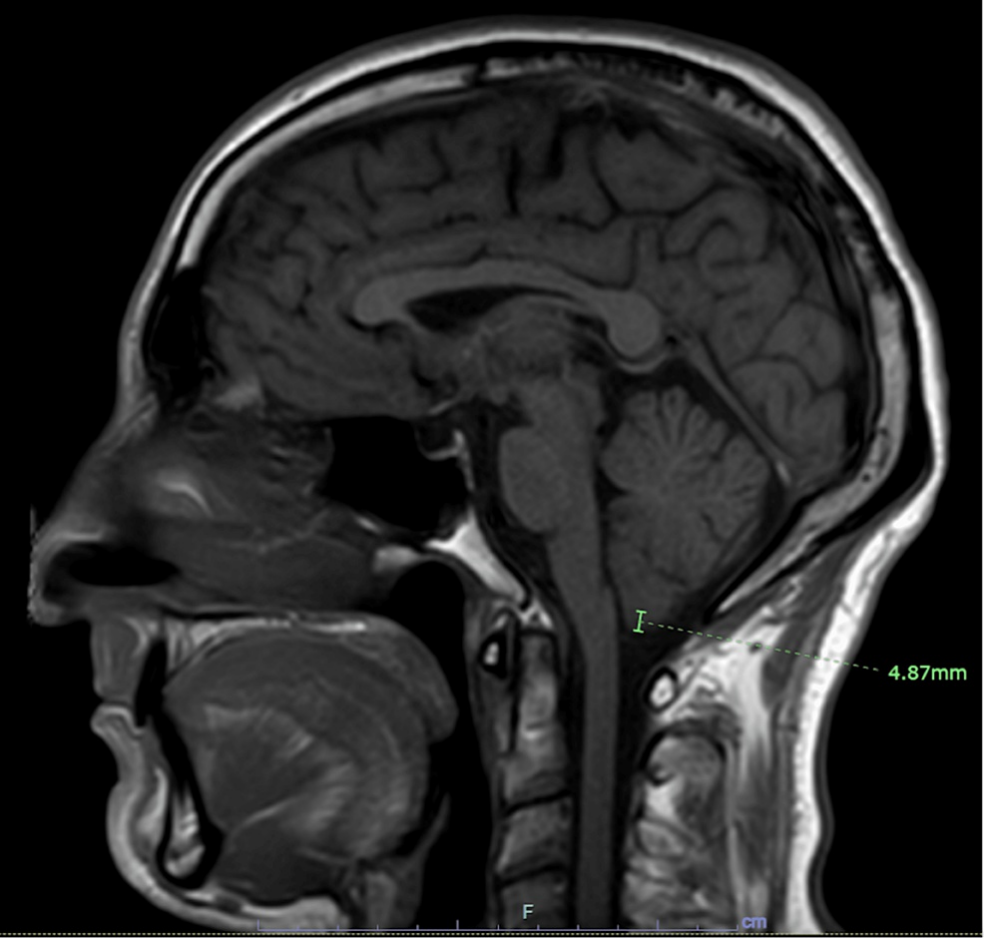
Non-Contrast Enhanced Sagittal T1-weighted Magnetic Resonance Image (MRI) of the Brain Demonstrates 4.87 mm Downward Herniation of the Cerebellar Tonsils into the Foramen Magnum

Non-contrast computed tomography (CT) of the head was negative for hemorrhage, herniation, mass, or hydrocephalus. Magnetic resonance venogram (MRV) was negative for infarction. MRI of the brain with and without contrast was positive for mild small vessel disease in the periventricular area, partially empty sella, and mild ectopia of bilateral cerebellar tonsils 4.87 mm below the foramen magnum with crowding (Figure 1). These MRI findings were consistent with CM1. Subsequently, he was evaluated using lumbar puncture to evaluate the intracranial pressure (ICP). The patient had an opening pressure 10 cm H_2_O, with cerebrospinal fluid (CSF) analysis showing clear and colorless fluid with 5 WBC/mm^3^, 250 RBC/mm^3^, glucose levels of 68 mg/dl, and protein levels of 42 mg/dl. These findings indicate normal intracranial pressure. Thus, this patient was diagnosed with Chiari I malformation, presenting with a rare symptomology of unilateral peripheral visual field defects and bilateral optic disk swelling with normal intracranial pressure. The patient was advised to follow up in the outpatient neurology and ophthalmology clinic.

## Discussion

The presentation of CM1 ranges from asymptomatic to severe with tinnitus, vertigo, ataxia, headache, myelopathy, neck pain, nausea, and vomiting [3]. It can also be associated with hydrocephalus, syringomyelia, sleep apnea, and scoliosis [3]. The true incidence of CM1 is unknown due to the asymptomatic nature of presentation in some patients; however, the estimated incidence is 0.5-3.5% of the general population [3]. Furthermore, the most common age group of presentation for CM1 is children to young adults. Although CM1 can be asymptomatic and may not require intervention, symptomatic patients may use acetazolamide to aid in reducing intracranial pressure. Patients with refractory increased intracranial pressure may have a serious risk of brain herniation and require neurosurgical intervention. Surgeries include foramen magnum decompressions, occipitocervical fixation, odontectomy, or cerebellar tonsillectomy [4]. 

This is a unique case of CM1 due to the atypical presentation of symptomatology of peripheral visual field defects with bilateral optic swelling in the absence of hydrocephalus or elevated intracranial pressure on the lumbar puncture. In addition, this is an uncommon age of presentation at 55 years old. Very few cases in literature document this sign in CM1. One case reported papilledema, bilateral anterior uveitis, and elevated ICP without hydrocephalus in a 16-year-old with CM1 [5]. Treatment with acetazolamide, prednisolone, and ophthalmic 1% cyclopentolate drops resulted in complete resolution of optic nerve swelling at the six-month follow-up [5]. In contrast, another case reports papilledema in a CM1 patient with concurrent idiopathic intracranial hypertension (IIH), making it unclear whether the papilledema originated secondarily to CM1 or IIH [6]. A case series involving 12 CM1 patients reported strabismus and diplopia [7]. They were managed with prism glasses and strabismus surgery. Three patients required neurosurgical decompression to prevent brain herniation. 

It is unexpected to observe optic disk swelling in the presence of normal intracranial pressure, such as in this patient with an ICP of 10 mmHg. Papilledema, defined as swelling of the optic disk, typically occurs due to increased pressure on the optic nerve resulting in edema, which can be visualized through fundoscopy. Increased pressure on the optic nerve could be from elevated intracranial pressure, or infiltrative or compressive processes, such as tumors [8]. In the literature, pseudopapilledema is reported as a cause of optic disk swelling without elevated intracranial pressure, originating from optic disk drusen (0.2-3.1% of cases), congenitally small nerves, optic nerve head crowding, and various tumors near the optic nerve [8]. One case reported this pathology in a 32-year-old CM1 patient due to optic drusen. Ultrasonography, optical coherence tomography, and intravenous fluorescein angiography (IFA) are used to diagnose pseudopapilledema and may be the next step in evaluating the patient in our report [8,9]. Anterior ischemic optic neuropathy may be considered; however, the patient in this case did not have any relevant medical history suggesting ischemic processes, such as diabetes or hypertension.

A partial empty sella sign may be an incidental finding. However, we must, at the least, consider this as a pathology prior to dismissing it. The patient in this case report possesses an empty sella sign with ophthalmic defects and a normal pressure on lumbar puncture, which may make normal pressure pseudotumor cerebri a potential diagnosis. This finding was characterized in a case series, describing an empty sella sign on MRI, an average lumbar pressure of 11 mmHg, and ophthalmic defects such as horizontal diplopia, bilateral abducens palsy, optic nerve swelling on fundoscopy, and an enlarged blind spot [10,11].

## Conclusions

The case involves an older patient presenting with bilateral optic disk swelling and peripheral visual field defects, without the typical symptoms of increased intracranial pressure or hydrocephalus as the initial presentation of Chiari I malformation. Diagnostic imaging confirmed mild cerebellar ectopia consistent with CM1. This presentation is especially paradoxical due to the presence of bilateral optic disk swelling with normal opening pressure on lumbar puncture. Future efforts should be made to document patients with atypical presentations of CM to help understand the range of symptomology and the true incidence of this pathology.
